# Effect of SDF-1 with biphasic ceramic-like bone graft on the repair of rabbit radial defect

**DOI:** 10.1186/s13018-019-1277-8

**Published:** 2019-07-22

**Authors:** Fuke Wang, Guiran Yang, Yu Xiao, Chuan He, Guofeng Cai, En Song, Yanlin Li

**Affiliations:** Department of Sports Medicine, The First Affiliated Hospital of Kunming Medical University, No. 4 Building, Xichang Road, Wuhua District, Kunming, 650032 Yunnan Province China

**Keywords:** Stromal cell-derived factor-1, Biphasic ceramic-like biological bone, Bone defect, Bone marrow mesenchymal stem cells, Bone tissue engineering

## Abstract

**Background:**

This study aimed to investigate the effects of stromal cell-derived factor-1 (SDF-1) on biphasic ceramic-like biologic bone (BCBB) in vivo on the repair of large segment bone defect in rabbits.

**Methods:**

A large-segment radius defect model of the rabbits was constructed. In the experimental group, BCBB with SDF-1 sustained-release system were implanted into the bone defect site. Other three groups including normal control, autologous bone graft, and BCBB implantation without SDF-1 were set. After surgery, general observation, X-ray radiography and scoring, and tissue section staining were performed at 2, 4, 8, 12, and 24 weeks post-implantation.

**Results:**

By general observation, X-ray radiography and grading and tissue section staining observation, we found that the BCBB carrying SDF-1 was better than those in the group of BCBB without SDF-1 (*P* < 0.05). BCBB scaffold had certain bone conduction capacity, and the BCBB scaffold carrying SDF-1 had improved bone conduction ability and possessed bone induction ability. In the case of carrying SDF-1, it can be used to repair large bone defects in a shorter time than simply using BCBB, which is equivalent to the effect of autologous bone.

**Conclusion:**

BCBB scaffold carrying SDF-1 can promote the repair effect on a large bone defect, which is equivalent to the effect of autologous bone.

## Background

Bone graft substitute materials are used by orthopedic surgeons to promote bone formation and repair bone defects [1, 2]. Compared with autologous bone materials and allogeneic bone materials, xenogeneic bone-derived implants have advantages such as wide sources, easy tissue preparation, and economic properties [3]. However, there are also potential disease transmission and common defects such as graft immune rejection [4]. Therefore, the xenograft bone should be treated with physical and chemical processing before use to minimize risks and to substantially decrease antigenicity [3, 5]. The ceramic-like xenogeneic bone formed by appropriate physical and chemical treatment and low-temperature calcination has a natural reticulated pore structure [6], and its main component is hydroxyapatite (HAP), while HAP is a slow-absorbing ceramic with poor biodegradability [7]. Therefore, it is often necessary to modify or compound with other materials to accelerate its absorption. At present, biologically derived materials related to bovine bone source have been developed and extensively studied [8, 9]. Lin et al. used the method of adding two sodium bovine calcinations and adding sodium pyrophosphate solution to convert HAP into tricalcium phosphate (TCP) with better degradation performance [5].

A previous study showed that after specific physical and chemical treatment of the pig cancellous bone, they prepared biphasic ceramic-like biologic bone (BCBB) mainly containing HAP and TCP, showing the certain osteogenic effect on the repair of radial bone defect model in animals [10]. It was found that this bone-derived BCBB of pig also had good biocompatibility and bone conduction ability [11]. In terms of physical and chemical properties, mineral inorganic salt Ca, P, organic protein content, and porosity of BCBB are similar to the human bone [11]. Therefore, BCBB can also be used as a heterogeneous bone-derived material with great potential, which is worthy of further research and exploration.

The chemokine stromal cell-derived factor-1 (SDF-1) has been widely investigated for its characteristic of homing bone marrow mesenchymal stem cells (MSCs) to the site of injury and inflammation [12, 13]. MSCs have been found to be responsible for tissue repair and immunological disorders by migration to the injury and inflammation in response to SDF-1 [13]. The SDF-1/CXCR4 axis is the most important chemotactic homing pathway on the surface of bone MSCs, and it is also the most widely used signal pathway in osteogenesis-related research [14]. The BCBB itself has a certain bone conduction capacity, and we speculate that the combination of BCBB and SDF-1 can achieve the effect of bone repair.

Currently, no other growth factors have been reported to possess the chemotaxis effect of bone MSC homing, comparable to SDF-1. Thus, in this study, the effect of carrying SDF-1 on repairing a large bone defect of BCBB was explored. Porcine bone was used as a raw material to prepare non-immunogenic BCBB by physicochemical processing. We expected that our findings could provide an animal experimental basis for clinical application in the future.

## Methods

### Animals

Forty 3-month-old healthy Japanese big-ear white rabbits of either gender (weighing 2–2.5 kg) and four 8-month-old healthy Diannan small-ear pigs of either gender (weighing 20 kg) were purchased from the Experimental Animal Center Department of Laboratory Animal Science, Kunming Medical University (Kunming, China).

All animal experimental procedures were approved by the Ethics Committee of Kunming Medical University.

### Preparation of SDF-1 sustained-release system

The commercially available chitosan (CS; 100 μg; Adamas Reagent Co., Ltd., Shanghai, China), 50 μl of 1% acetic acid solution, and 4850 μl of double distilled water were added to a 50-ml beaker to prepare a CS buffer solution. The beaker was covered with tin foil paper, and the CS was completely dissolved by stirring at low speed for 2 h at 200 rpm on a magnetic stirrer at room temperature. Then, 2 μg of SDF-1 (PeproTech Inc., Rocky Hill, New Jersey, USA) was added into the CS slow-release solution; and the mixture was stirred at a low speed of 200 rpm for another 2 h on the magnetic stirrer to obtain a CS/SDF-1 sustained-release system.

### Preparation of BCBB

This process was performed as previously described by Xiu et al. [15] with modifications. In brief, all the procedures were carried out under sterile conditions. The femur and tibia of Diannan small-ear pigs were washed and disinfected, and the bone saw was also disinfected. The femur was cut horizontally with the bone saw at the lesser trochanter. The femur was cut horizontally with the bone saw on the medial and lateral iliac crests below the femur, and the femur in the middle of the femur was discarded, leaving the greater trochanter, lesser trochanter, and medial and lateral iliac crest. In addition, the tibia was cut horizontally with the bone saw at the tibial trochanter, and the lower part of the tibia was discarded, leaving the upper part. Subsequently, the femur and tibia bones were cut into 0.5-cm thick bone pieces in the horizontal direction with the bone saw. The cortical bone was removed along the edge of the bone with the bone saw. The cancellous bone piece from which the cortical bone was removed was sawed into a 1.5 cm × 0.5 cm × 0.5 cm cuboid bone piece with the bone saw. Then, the bone pieces were put into a 500-ml beaker which was added with a small amount of double distilled water and then boiled for 6 h; then, the cancellous bone pieces were put into a porcelain boat that was subsequently put into a desiccator (Dafeng Laboratory Equipment Co., Ltd., Shanghai, China) for overnight. The porcelain boat with cancellous bone pieces was put into a muffle furnace (Luoyang Liyu Furnace Co., Ltd., China) and raised to 800 °C at 10 °C/min and held for 1 h.

The calcined cancellous bone pieces were then soaked with 0.04 mol/ml sodium pyrophosphate solution in a glass test tube, being ultrasonically vibrated at 40 KHz for 30 min and let the sodium pyrophosphate solution entering the cancellous bone void. After that, the cancellous bone was put into the porcelain boat which was then put into the desiccator overnight. The porcelain boat with the cancellous bone was put into the muffle furnace and raised to 1150 °C at 10 °C/min and held for 1 h to obtain BCBB. After cooling, the BCBB was put in the desiccator and prepared for use.

### SDF-1 loading onto BCBB

All the procedures were carried out under sterile conditions. The BCBB was taken out of the desiccator and placed into a glass test tube with CS/SDF-1 slow-release solution to ensure that the BCBB was completely immersed in the CS/SDF-1 sustained-release solution. The glass tube mouth was sealed with sealing glue, and the tube was ultrasonically vibrated at 40 KHz for 30 min, so that CS/SDF-1 slow-release system was loaded on the inner wall of BCBB void. The BCBB was removed from the glass test tube with sterile forceps and placed on an aseptic table to dry at room temperature. After drying, the BCBB was transferred into a 2-ml centrifuge tube that was then sealed and put into the desiccator for later use.

### Radius defect model in rabbits

**Fig. 1:**
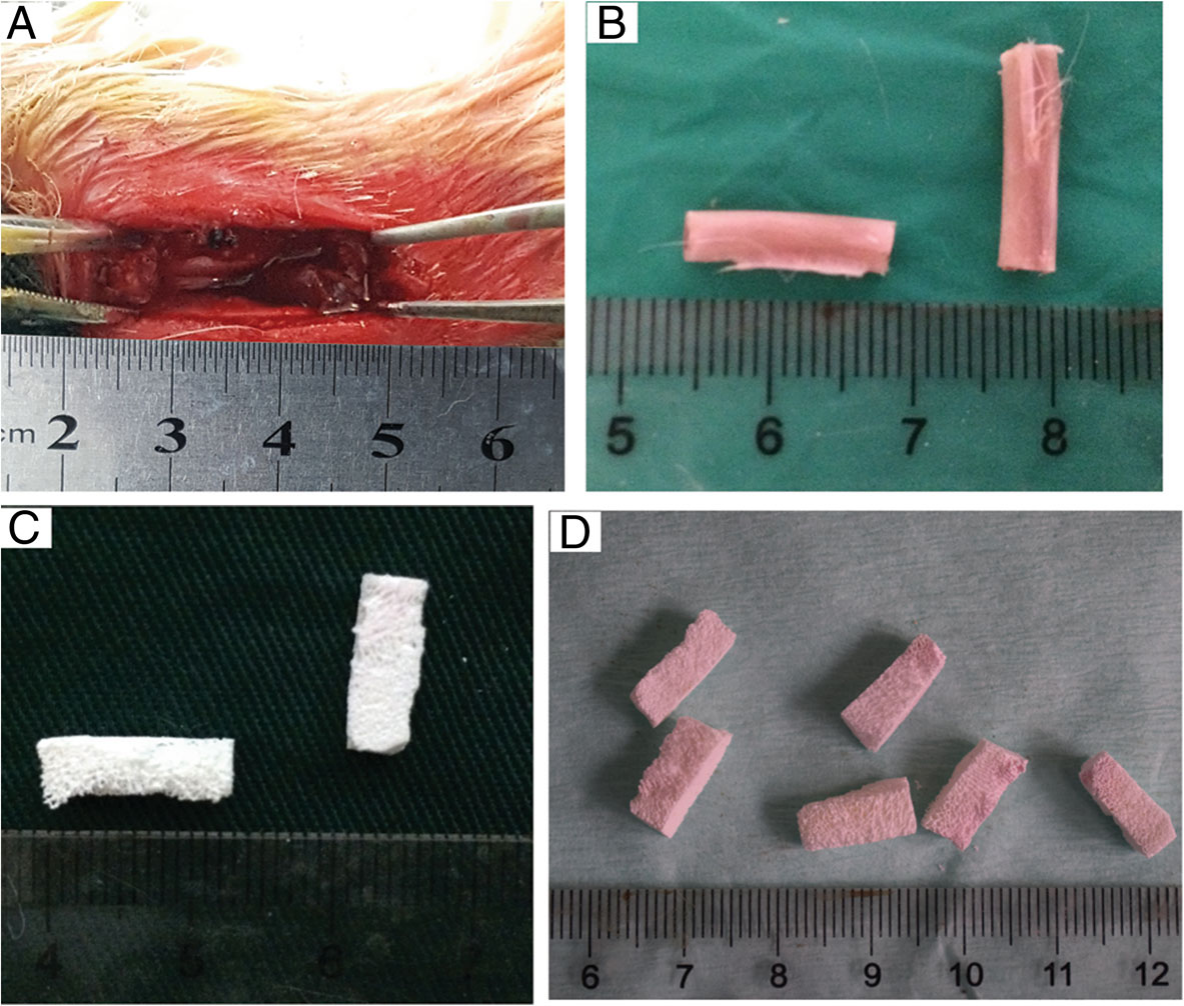
Construction of rabbit radial defect model and biphasic ceramic-like biologic bone (BCBB) carrying stromal cell-derived factor-1 (SDF-1). **a** A 1.5-cm radius defect. **b** Autogenous bone graft length. **c** The obtained BCBB was pale and ceramic-like. **d** The BCBB/SDF-1 composite bone was yellowish in color

The 40 healthy Japanese big-ear white rabbits were randomly divided into A (blank group), B (autologous bone group), C (BCBB group), and D (SDF-1/BCBB) groups (*n* = 10 in each group). All the procedures were performed under strict aseptic conditions. Rabbits in each group were anesthetized by an intraperitoneal injection of 10% chloral hydrate (3 ml/kg). After anesthesia, the rabbit was fixed on the operating table of the animal, and the rabbits’ fur was excised from the front limb, and the surgical site was exposed, followed by disinfection. A 1.5-cm incision in the middle part of the radius of the forelimb was made using a scalpel, and the tissue overlying the mid-shaft of the radius dissected. The periosteum of the bone surface was peeled off with a periosteal stripper, and a scalpel was used to mark about 0.8 cm at both ends of the midline of the radius. The length of the bone section was measured with a stainless steel ruler to ensure a 1.5-cm radius defect (Fig. 1a). In the marked position of the scalpel, the bone puncture needle was used for grinding, the bone was slowly cut off along the grinding place with the bone nippers, and the broken end was ground down with the bone file. The length of the bone that removed was about 1.5 cm (Fig. 1b).

The surgical field was rinsed with hydrogen peroxide and iodine volt. The radial bone was removed after bilateral radial truncation in group A. Group B was implanted with autologous contralateral radius on both sides. Group C was implanted bilaterally with BCBB. Group D was implanted bilaterally with BCBB/SDF-1, and the implantation of the implant was ensured. The incision was sutured layer by layer; the skin was sutured intermittently and covered with sterile gauze. Daily intramuscular injection of 400,000 units of penicillin was performed 3 days after the operation to prevent wound infection, and then, the penicillin was used once every other day to observe the wound healing.

### Observations

#### Gross observation

The surgical site, activities, and eating status of the animals in each group were observed postoperatively. Two rabbits of group A, B, C, and D were sacrificed by air embolism at 2, 4, 8, 12, and 24 weeks postoperatively to observe the wound healing, changes of implants, and the relationship between implants and the surrounding bone and tissue.

#### Radiologic evaluation

Two rabbits in groups A, B, C, and D were sacrificed at 2, 4, 8, 12, and 24 weeks postoperatively. The orthotopic X-ray of the ulna and radius of the forelimb were photographed to detect the repair effect of large segmental bone defects. X-ray films were scored according to Lane-Sandhu grading [16]; the scores were statistically analyzed using SPSS.17.0.

#### Histology

Two rabbits of groups A, B, C, and D were sacrificed at 2, 4, 8, 12, and 24 weeks postoperatively. The radius was cut at 0.5 cm at both ends of the implant, and the specimen was completely intercepted and fixed with 75% ethanol for 15 days. After soaking in 85% alcohol for 12 h, 90% alcohol for 24 h, 95% alcohol for 24 h, absolute ethanol for 24 h, and pure toluene for 6 h for gradient dehydration, the specimen was put into the embedded container with a few block copolymers of poly(methyl methacrylate) (PMMA) and then PMMA prepolymer solution was added, followed by vacuuming for 5 h, so that there was no bubble in the specimen. The embedding container was completely enclosed and let it stand in the refrigerator at 4 °C for 14 days. Polymerization was done at room temperature until PMMA was fully hardened. The fixed gross specimen was put into the low-speed precision cutting machine, and the cutting thickness was adjusted to 150 mm for slicing. Then, 1 ml of polylysine was added to the slides, and the sections were attached to a glass slide and fixed by extrusion for 24 h, and air bubbles were discharged. The sections were polished to 50 μm for subsequent use.

The sections were stained with Van Gieson and hematoxylin-eosin (H&E) stains according to standard protocols. The histological observation was performed using an Olympus microscope (Olympus, Tokyo, Japan).

#### Measurement of bone area

Two slices were randomly selected from group B, C, and D, respectively. Two fields of view at both ends and middle part of the bone defect were taken; a total of 12 visual fields were measured using a phase contrast microscope (BX53; Olympus, Tokyo, Japan) under × 40 magnifications. The bone areas were calculated with the histogram function of Photoshop 11.0 software [17, 18].

#### Statistical analyses

All experimental data were represented as mean ± standard deviation (SD). One-way analysis of variance (ANOVA) was used to analyze the results of multiple groups of statistical data using SPSS 17.0, and *t* test was performed between two groups. *P* < 0.05 was statistically significant.

## Results

### Gross observation

The obtained BCBB is pale, ceramic-like, with a large number of pores visible on the surface (Fig. 1c). The BCBB/SDF-1 composite bone was yellowish in color and had a large amount of pores filled with CS/SDF-1 (Fig. 1d).

On the first day after the operation, the animals were able to move and eat normally, and there was no obvious exudation at the wound which was wrapped with gauze. At the time of dressing change, scabs were visible and healed well. Within 1 week after surgery, the forearm was slightly swollen compared to the healthy side and gradually subsided 3 days later. Two weeks after the operation, a part of the scab exfoliated and the skin healed well. The hair growth at the surgical site 1 month after the operation was not significantly different from the surrounding non-surgical area, and the skin scar was not obvious. No sign of surgical site infection was found during the experiment.

### Observation at 2 weeks after operation

**Fig. 2:**
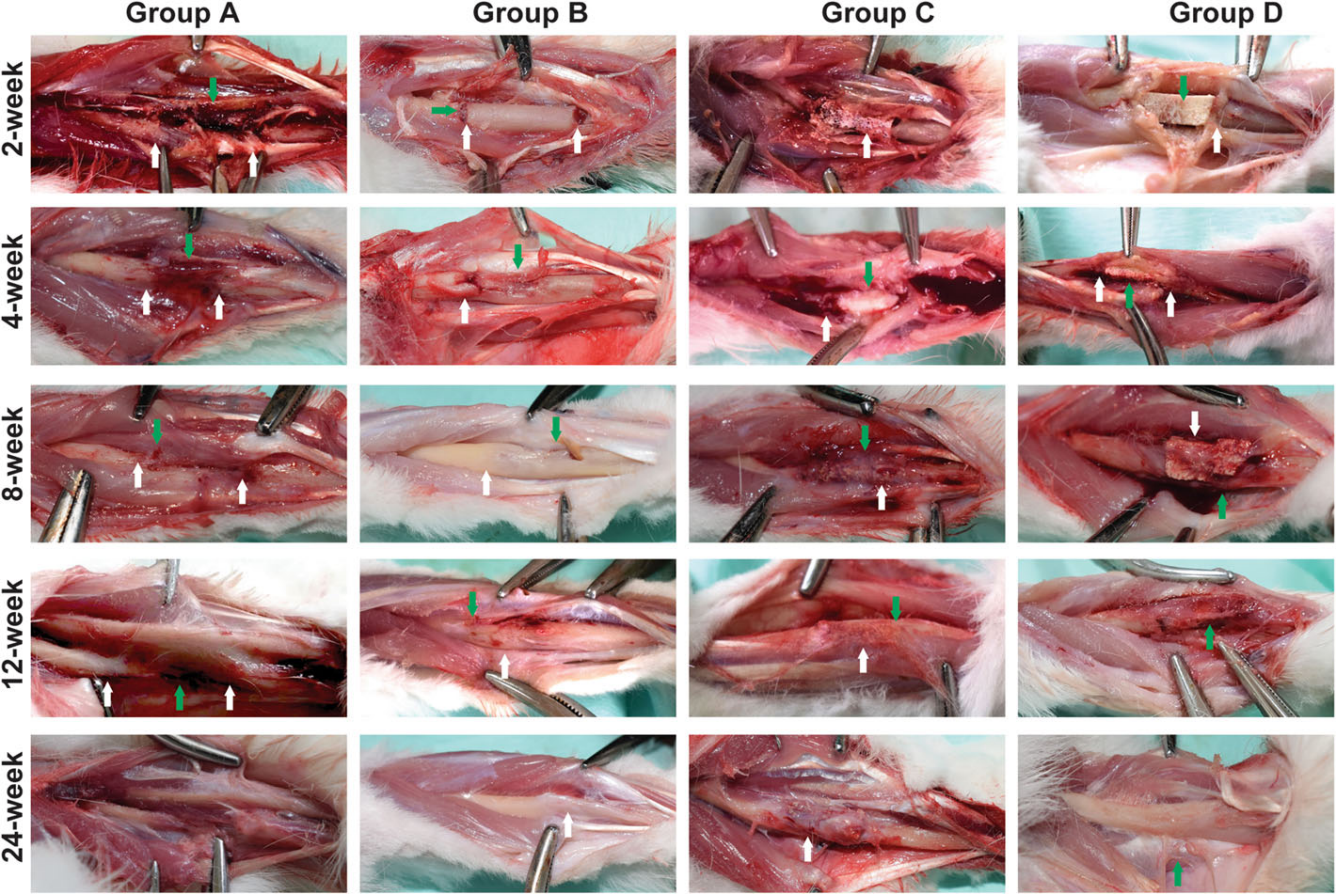
Gross observation post-implantation. Group **a** at the 2nd week: the bone defect was obvious (white arrow), massive tissue fluid exudation, and formation of fresh bone tissues (green arrow); there are no signs of infection. Group **b** at the 2nd week: the bone stump neat (white arrow); massive fibrous tissue formed in the gap (green arrow). Group **c** at the 2nd week: the boundary between the BCBB and the bone stump was clear, with fibrous tissue filling and no obvious signs of infection (white arrow). Group **d** at 2nd week: there was no significant difference between group **d** and group **c**, the structure of the material was complete, the gap between the host and the material was filled with fibrous tissue (white arrow), there was new tissue ingrowth in the pores of the material (green arrow), and no obvious signs of infection. Group **a** at the 4th week: the exudation was reduced and the medullary cavity was closed by fibrous tissue (white arrow) and fibrous tissue adhesion (green arrow). Group **b** at the 4th week: at the bone stump, formed the fibrous callus (white arrow) and periosteal tissue formed on the surface of the autologous bone (green arrow). Group **c** at the 4th week: the surface of the material was complete, the host bone tissue is closely connected (white arrow), and the callus wrapped around the surface of the material (green arrow). Group **d** at the 4th week: the surface structure of the material was complete, and the material was closely connected with the host bone (white arrow), surrounded by the callus, rich in blood supply (green arrow). Group **a** at the 8th week: the medullary cavity was closed by the bone tissue and surrounded with a large amount of fibrous tissue and muscles (white arrow), and the exudation was reduced and absorbed (green arrow). Group **b** at the 8th week: the callus becomes bony union (white arrow), and the growth of the callus and the resorption and reconstruction of the bone graft were obvious (green arrow). Group **c** at the 8th week: the material contour was complete, and the BCBB was closely connected to the host bone (green arrow), the surface of the material and the pores were filled with callus, and the material was absorbed and degraded in different degrees (white arrow). Group **d** at the 8th week: the material structure is not as complete as 4 weeks, the surface can be seen a large number of callus formation (white arrow), the material and the host bone tightly connected, and the material is absorbed; repair section had a good shape (green arrow). Group **a** at the 12th week: bone resorption in the area of the bone defect (black arrow), the surrounding fibrous tissue formed fibrous scar (green arrow). Group **b** at the 12th week: the autogenous bone was completely fused with the original bone (white arrow), the surface was covered with periosteum-like tissue, and the blood supply was abundant (green arrow). Group **c** at the 12th week: materials and bone tissue boundary are not clear (green arrow), and the repair section had a good shape (white arrow). Group **d** at the 12th week: the material and the bone formation are firm, the boundary is not clear, the material was partially absorbed, the new bone has grown, and the partial shape approaches the normal radius (green arrow). Group **a** at the 24th week: there was no significant difference between 12 and 24 weeks of observation. Group **b** at the 24th week has the same shape close to the radius in 24 weeks (white arrow) and 12 weeks. Group **c** at the 24th week: the surface of the material is rich with callus, rich in blood supply, and the boundary between the host bone tissue is not clear and the repair section had a better shape than 12 weeks (black arrow). Group **d** at the 24th week: the repair section was well shaped, and there was no significant difference between the host bone and material (green arrow)

In each group, the fracture ends of the bone were visible. A large amount of tissue fluid exudation and new tissue formation occurred in the fracture ends of group A. A large number of new fiber-like tissues were seen in the gap between autologous bone and host bone in group B. The BCBB materials in group C and group D were clear, and there was tissue growth in the space between the materials (Fig. 2).

### Observation at 4 weeks after operation

In group A, the exudation was reduced, the medullary cavity was closed by fibrous tissue, and the surrounding tissue formed fibrotic adhesion. In group B, new fibrous callus wrapped around the broken end of the bone and periosteum-like tissue was found on the surface of the autogenous bone. In group C, the surface of the material was intact, closely bound with the host bone tissue, and the new callus wrapped around the surface of the material. In group D, the surface structure of the material was intact, tightly bound to the host bone, forming new callus around and abundant blood supply (Fig. 2).

### Observation at 8 weeks after operation

In group A, bone fractured ends were closed, and a large number of fibrous tissues were wrapped, which made it difficult to separate from the surrounding tissues and muscles, and fibrous scars were formed. In group B, the autogenous bone and host bone formed bone healing, callus growth was obvious, and bone graft was absorbed and remodeled. The material of group C was complete and tightly bound to the host bone, the surface and pores of the material were filled with tissues, and the BCBB material was absorbed and degraded to different extents. The BCBB material structure of group D was relatively complete, with a large number of calluses forming on the surface and the material is closely bound to the host bone, with the material absorption, and the repair section had a good shape (Fig. 2).

### Observation at 12 weeks after operation

In group A, bone resorption occurred at the defect site, and the surrounding fibrous tissue formed scar adhesion. In group B, the autogenous bone was completely fused with the host bone, and the surface of the bone was covered with periosteum-like tissue, and the blood supply was abundant; in group C, the graft materials of group C were not clearly separated from bone tissue, forming a firm union, and the repair segment was well shaped. In group D, the material and bone formed a firm union with unclear boundaries, graft materials were absorbed, and the shape of some was close to the normal radius bone (Fig. 2).

### Observation at 24 weeks after operation

There was no significant difference between the visual observation at 24 weeks and 12 weeks in group A. The shape of group B at 24 weeks was similar to the radius. In group C, the surface of tissue was packed tightly, the blood supply was abundant, the boundary between the bone tissue and the host bone was unclear, and the repair segment had a good shape. The repaired segment of group D was well shaped and showed no obvious difference from the host bone shape (Fig. 2).

### Radiologic outcomes

**Fig. 3:**
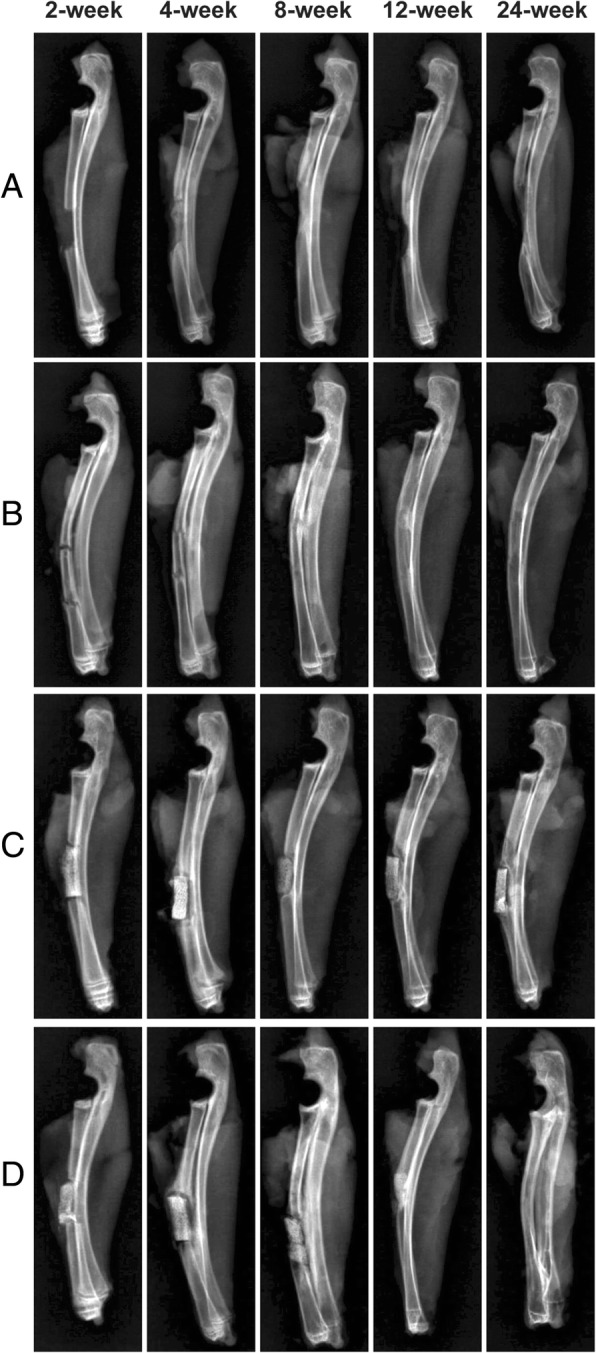
The X-ray results of four groups at 2, 4, 8, 12, and 24 weeks post-implantation. The X-ray results of group **a**, at each time point, the area of the bone defect was observed. With the passage of time, the end of the fracture was gradually absorbed and hardened. The X-ray results of group **b**, autogenous bone and the bone stump had good involution. In the 8th week, the broken area is distinguished. In the 12th and 24th weeks, the implanted bone was absorbed and the morphology was similar to that of the radius. The X-ray results of group **c**, the material and the bone stump had good involution. In the 4th week, callus wraps around the surface of the material. In the 8th week, the material was absorbed and degraded in different degrees. In the 12th week, the new bone appeared in some parts, and the material was tightly bound to the host bone for 24 weeks. The X-ray results of group **d**, the material was well connected with the host bone, and 4 weeks later, it was closely connected with the host bone. The bone density increased in the 8 weeks, and the new bone tissue appeared at the end of the 12th week. In the 24th week, the material was similar to the radius

In group A, the bright zone of the bone defect was observed at every time point. At 2 weeks, the bone defect was obvious and transparent, the fibrous tissue was filled, the fracture margin was neat, and the medullary cavity was connected with the outside. At 4 weeks, a small amount of bone absorption and remodeling appeared at the fracture end, and the fibrous tissue filled the defect area and filled the medullary cavity. At 8 weeks, the bone absorption and remodeling at the fracture end was obvious, the bone absorption at the proximal ulnar side was less, and the bone absorption at the distal ulna side was more; high smoothness and brightness at the broken end was found, the fibrous scar was filled in the defect area, and the medullary cavity was closed. At 12 weeks, the bone cortex of the fracture end was hardened and smooth, and the X-ray changes were not obvious at 24 and 12 weeks (Fig. 3).

In group B, at 2 weeks, the interspace between the broken end and the implanted bone was clear, the medullary cavity was exposed, and the fibrous tissue was proliferated. At 4 weeks, the fibrous tissue was proliferated in the interspace between the broken end and the implanted bone, the boundary between the autogenous bone and the broken bone was blurred, and a small amount of proliferative bone-like tissue was filled in the medullary cavity of the broken bone. At 8 weeks, the bone and the implanted bone formed a bone connection, and the bone cortex was thickened at the bone junction, the fibrous tissue was wrapped, and the medullary cavity structure was formed. At 12 weeks, the broken end of the bone was continuous with the implanted bone cortex, and the medullary cavity was connected, the brightness of the cortical bone was increased at the junction, and the shape was rough. At 24 weeks, the bone cortex was continuous and intact, and the medullary cavity was reconstructed. There is no difference with the normal radius (Fig. 3).

In group C, at 2 weeks, the implanted BCBB graft material was well aligned with the fracture end without obvious displacement, the material was in complete shape, the internal pore structure was clear, the gap between the fracture end and the host bone was clearly visible, and the medullary cavity was exposed without obvious callus growth. At 4 weeks, the broken end bone showed different degrees of absorption and remodeling, the edge of the implanted material was slightly blurred, and the general structure was complete. The surrounding hyperplastic fibrous tissue was wrapped around it. At the 8th week, the edge of the implant material showed different degrees of degradation and absorption, the broken end of the marrow cavity was not closed, and bone callus filled the cavity. At 12 weeks, the density of graft materials increased, showing a high-brightness shadow, which was similar to the density of radius, and partly surrounded the new bone. At 24 weeks, the graft material density increased, the density of the connection site was similar to that of the radius, the morphology became narrower, and the connection was close to the bone (Fig. 3).

In group D, at 2 weeks, the implants were well aligned with the broken ends, without obvious displacement, with clear pore structure, clear space between the broken ends and the host bone, and exposure of the medullary cavity. At 4 weeks, the material had a complete appearance, the density of the contact surface with the broken bone was increased, the bone was remodeled, and the material was covered by the bone callus. At 8 weeks, the graft and the host bone were not clearly separated, the density was similar to the host bone, the pore structure in the graft was not clear, and the medullary cavity was not closed. At 12 weeks, the graft material was partially absorbed, shortened, and narrowed, and bone healing was shown at the fracture end, the density of repaired area was similar to that of radius, and the boundary was unclear. At 24 weeks, the medullary cavity was reconstructed. The cortex of the repaired area was continuous and smooth, similar to that of the radius, and the density of the medullary cavity was slightly higher, which was the residual shadow of the material (Fig. 3).

### X-ray score

The X-rays were scored according to the X-ray scoring standard of Lane et al. [16]. The evaluation contents were three parts: the healing of the graft and the host bone, the formation of the marrow cavity, and the degree of reconstruction of the bone marrow cavity. The results showed that there were differences between group B and group C at 8, 12, and 24 weeks (*P* < 0.05). There was a difference between group B and group D at 8 weeks (*P* < 0.05). There was a difference between group C and group D at 24 weeks (*P* < 0.05). The B group had the best bone defect repair effect, the D group was the second, and the C group was the worst. There was no significant difference in the X-ray results of the bone repair between group B and group D after 12 weeks (Table 1).

**Table 1:** The X-ray score of the four groups at different time

	2 weeks	4 weeks	8 weeks	12 weeks	24-week
Group A	0 ± 0	0 ± 0	0 ± 0	0 ± 0	0 ± 0
Group B	0 ± 0	2.33 ± 1.54	6.33 ± 0.58^*^	7.33 ± 1.53	10.33 ± 1.53
Group C	0 ± 0	2.00 ± 1.00	3.67 ± 0.58^*^	5.00 ± 1.00^*^	5.33 ± 1.53^*^
Group D	0 ± 0	2.33 ± 0.58	4.00 ± 1.00^#^	6.33 ± 0.58	8.67 ± 1.12^□^
*F*	–	0.125	11.400	3.364	9.722
*P* value	–	0.885	< 0.05	0.105	< 0.05

Data are expressed as mean ± standard deviation. There was no difference in the 2nd week for each group, which was not included in the statistical category. Group A had no difference in each time point and was not included in the statistical category. *P value < 0.05, group C compared with group B. ^#^*P* value < 0.05, group D compared with group B. □*P* value < 0.05, group C compared with group D

### Van Gieson staining

**Fig. 4:**
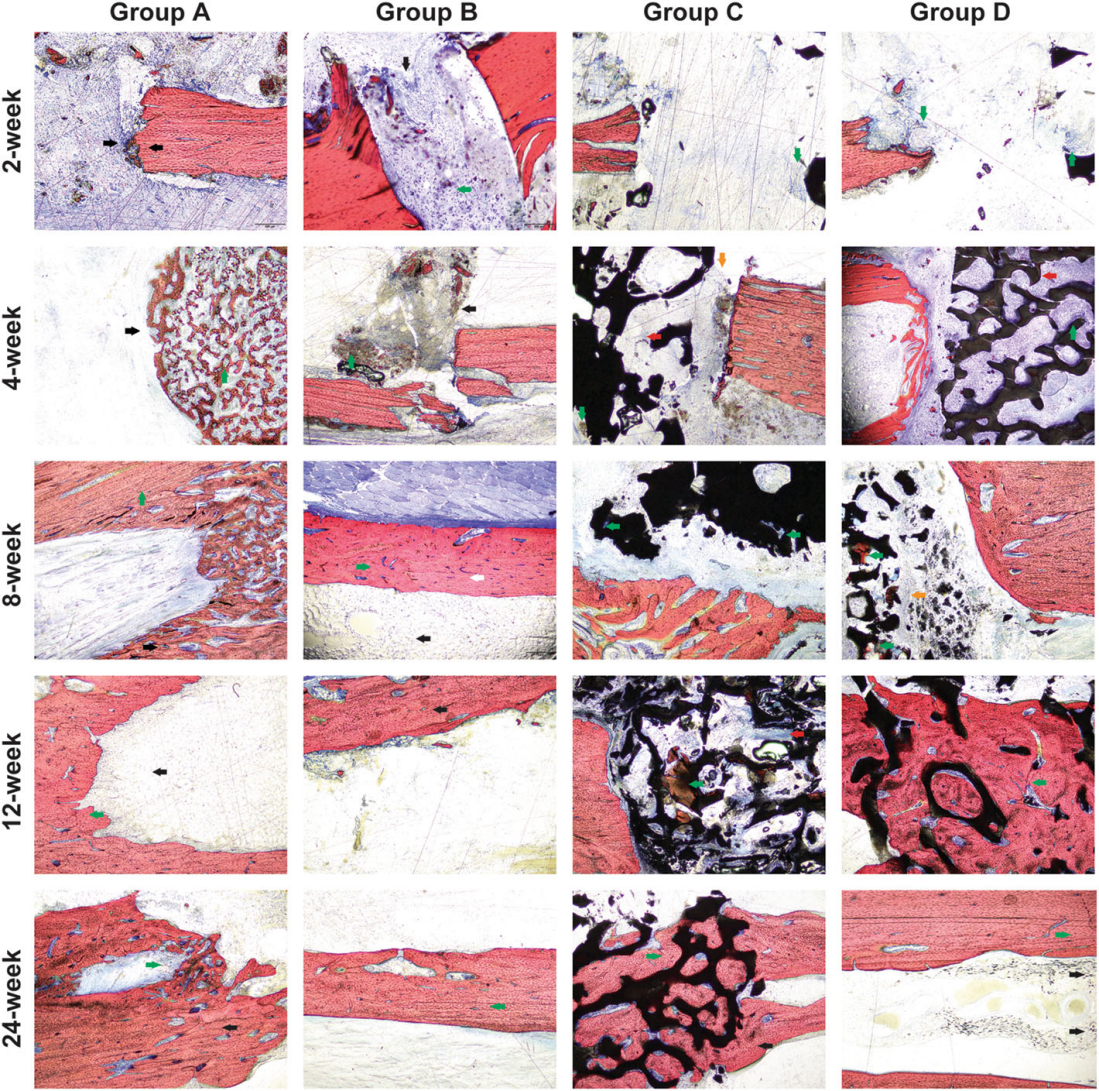
Van Gieson staining. Group **a** at the 2nd week: the bone stump was neat, the fibrous tissue proliferated, multinucleated cells were seen around (black arrow), the cell was infiltrated, and the bone was absorbed; no obvious signs of infection (LM × 40). Group **b** at the 2nd week: the fibrous tissue was connected with the host bone (black arrow), a large number of cells were infiltrated (green arrow), and no obvious signs of infection (LM × 40). Group **c** at the 2nd week: neovascularization and fibrous tissue grew into the material were less than that in group **d** (green arrow), the bone stump was neat, and there were no obvious signs of infection (LM × 40). Group **d** at the 2nd week: neovascularization and fibrous tissue grew into the material, a large number of cells were infiltrated (green arrow), and no obvious signs of infection (LM × 40). Group **a** at the 4th week: bone resorption and remodeling (green arrow) were observed around the bone stump (black arrow), and VG staining showed that large bone formation areas gradually closed the medullary cavity (black arrow) (LM × 40). Group **b** at the 4th week: the autogenous bone was closely connected with the host bone (black arrow), surrounded by a large number of blue fibrous callus (black arrow) and red stained bone callus (green arrow) (LM × 40). Group **c** at the 4th week: more blood vessels and fibrous tissue grew into the material (red arrow), there was a little bone formation (green arrow), and there was a fibrous connective tissue between the material and host bone (yellow arrow) (LM × 40). Group **d** at the 4th week: more blood vessels and fibrous tissue grew into the material, the new bone tissue was found in the pores (red arrow), and collagen was distributed along the surface of the material (green arrow) (LM × 40). Group **a** at the 8th week: the bone texture is clear in the bone stump area (black arrow), and the orientation of the trabecular bone in the area of bone resorption and remodeling section was irregular (green arrow) (LM × 40). Group **b** at the 8th week: the autologous bone marrow cavity is recanalized (black arrow), the orientation of bone trabecula was irregular (green arrow), and there was an obvious difference between the host bone and autologous bone (white arrow) (LM × 40). Group **c** at the 8th week: the collagen was formed on the surface of the material (green arrow) (LM × 40). Group **c** at the 8th week: the collagen was formed on the surface of the material (red arrow) (LM × 40). Group **d** at the 8th week: the new bone increased gradually from the two ends of the defect into the material, there was a red staining of the bone tissue (green arrow) and cell distribution along the surface of the material (yellow arrow) (LM × 40). Group **a** at the 12th week: the medullary cavity closure (black arrow) and bone continuity were good (green arrow) (LM × 40). Group **b** at the 12th week: autologous bone marrow cavity is recanalized, visible bone resorption and reconstruction were observed, the orientation of bone trabecula was regular (black arrow) (LM × 40). Group **c** at the 12th week: in the pores, the number of bone formation tissue was increased (green arrow), along the surface of the material distribution, increased blood vessels, and collagen deposition around the blood vessels (red arrow) (LM × 40). Group **d** at the 12th week: the number of the new bone was increased than before, the material occupied was reduced, the orientation of bone trabecula was irregular, the osteoblasts were active (green arrow), and the material and new bone were connected closely (LM × 40). Group **a** at the 24th week: multinucleated cell aggregation around the medullary cavity of bone adjacent side (green arrow), bone resorption and remodeling; the orientation of bone trabecula was regular (black arrow) (LM × 40). Group **b** at the 24th week: the cortical bone structure was clear, and the orientation of bone trabecula was regular (green arrow) (LM × 40). Group **c** at the 24th week: the materials’ occupied space was reduced, the pores were filled with new bone tissue (green arrow), the material and new bone were connected closely (black arrow), and the orientation of bone trabecula was irregular (LM × 40). Group **d** at the 24th week: all the materials were absorbed, the orientation of bone trabecula was regular, the calcification was good, and the reconstruction of the medullary cavity was complete (black arrow) (LM × 40)

At 2 weeks, the fractured ends of each group were obvious. In group A, the fractured ends were neat, the surrounding fibrous tissue proliferated, the cells infiltrated, and the multinucleated cells were seen around the broken ends of the bone, which were involved in bone resorption. No obvious inflammatory cell infiltration was observed. In group B, the fibrous tissue was connected between the autologous bone and the host bone. A large number of cells infiltrated without obvious inflammatory cell infiltration. In group C, the new blood vessels and fibrous tissue grew into the graft material, less than group D; bone fracture ends are neat without inflammatory cell infiltration. In group D, new blood vessels and fibrous tissues grew into the material, and a large number of cells were infiltrated, without inflammatory cell infiltration (Fig. 4).

**Fig. 5:**
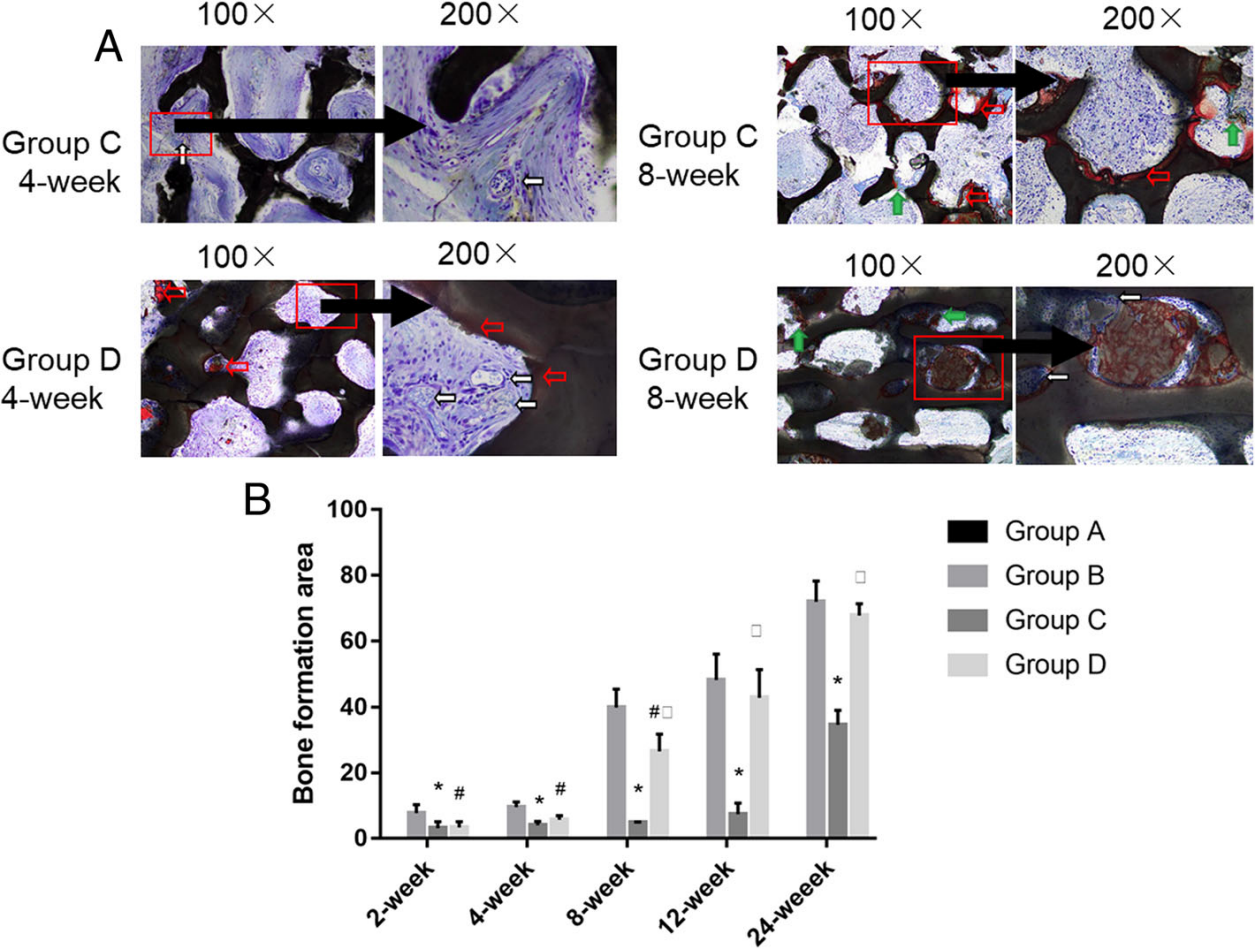
Van Gieson staining of C than D groups at × 100 and × 200 magnification and histogram of bone formation comparison of four groups. **a** Group C at the 4th week: more blood vessels and fibrous tissue grew into the material, and the new bone tissue was found in the pores (red arrow) (LM × 100). Group C at the 4th week: more blood vessels (white arrow) and fibrous tissue grew into the material, and the new bone tissue was found in the pores (red arrow), no bone formation (LM × 200). Group D at the 4th week: more blood vessels and fibrous tissue grew into the material, and the new bone tissue was found in the pores (red arrow) (LM × 100). Group D at the 4th week: more blood vessels (white arrow) and fibrous tissue grew into the material, and the new bone tissue was found in the pores (red arrow) (LM × 200). Group C at the 8th week: the collagen was formed on the surface of the material (green arrow), red stain osteoid like tissue on the surface of the material (red arrow) (LM × 100). Group C at the 8th week: the collagen was formed on the surface of the material (green arrow), red stain osteoid like tissue on the surface of the material (red arrow) (LM × 200). Group D at the 8th week: the new bone increased gradually from the two ends of the defect into the material, and there was a red staining of bone tissue (green arrow) (LM × 100). Group D at the 8th week: osteoblast cell was large and dark color, the core of the mesenchymal cell is shallow, has multiple projections, and is along the edge of the material distribution (white arrow) (LM × 200). **b** Histogram of bone formation comparison of four groups. *P value < 0.05, group C compared with group B. ^#^*P* value < 0.05, group D compared with group B. ^□^*P* value < 0.05, group C compared with group D

At 4 weeks, the bone around the broken end of group A was absorbed and remodeled, and the medullary cavity was gradually closed. The autologous bone of group B was tightly bound to the host bone, and there were a large number of the fibrous callus mixed with red-stained bone callus. In group C, more blood vessels and fibrous tissue grew into the graft material and a small amount of bone formation occurred, and fibrous tissue connection between the material and host bone was observed (Fig. 5). More blood vessels and fibrous tissue in the D group grew into the graft material (Fig. 5), the new bone tissue was seen in the graft material, and the collagen was distributed along the surface of the material (Fig. 4).

At 8 weeks, in group A, the bone trabeculae of broken ends had a clear texture, and the bone trabeculae in bone resorption and remodeling areas were disordered. In group B, the autologous bone marrow cavity was recanalized. The texture of the new trabecular bone was not well arranged, which was distinct from the host bone. Osteoblasts formed blue-stained collagen on the surface of the graft material and red-stained bone-like tissue appeared on the surface of the material. In group C, formed bone cells formed blue-stained collagen on the surface of the material (Fig. 5). In group D, new bone increased and gradually grew from both ends of the defect. Red staining bone tissue appeared in the graft material (Fig. 5). The osteoblast cells were large and dark in color, and the mesenchymal nuclei were shallow, and many protrusions were distributed along the edge of the material (Fig. 4).

At 12 weeks, in group A, the medullary cavity was closed and the bone was continuous. In group B, the autologous bone marrow cavity was recanalized. The medullary cavity was absorbed and reconstructed, and the trabecular bone texture was regular in the repaired area. In group C, the red staining bone tissue was increased along the material surface, the blood vessels were increased, and there was collagen deposition around the blood vessels. In group D, new bones are increased, the graft material space decreased, the trabecular arrangement of new bone was disordered, the cellular osteogenesis was active, and the graft material was bonded to the new bone.

At 24 weeks, in group A, the aggregation of polynuclear cells was found along the sclerotin in the medullary cavity side, bone resorption and remodeling was found, and bone trabecula texture was observed. In group B, the cortical bone structure was clear and trabecular bone was arranged regularly. In group C, the graft material occupied space was reduced and filled with new bone tissue (shown by the green arrow), and the bone binding between the material and new bone tissue (shown by the black arrow) was more disorderly. All materials in group D were absorbed, the new bone trabeculae were arranged in order, calcification was good (shown by the green arrow), the medullary cavity was reconstructed, and there was occasional residual material degradation outside the bone cortex of the lateral medullary cavity.

### Measurement of bone area

There were differences in bone formation at the same time point (*P* < 0.05) in each group, with B group constituting the most bone formation, followed by D group, and C group the least. There was no significant difference in bone formation between group C and group D before 4 weeks (*P* > 0.05), and all of them were less than group B. After 8 weeks, the bone formation in D was more than that in group C (*P* < 0.05), and there was no significant difference between group B and D after 12 weeks (*P* > 0.05). It can be considered that the final bone formation effect of group D was equivalent to that of group B and significantly superior to group C (Fig. 5b, Table 2).

**Table 2:** The bone formation area in different groups at different time

	2 weeks	4 weeks	8 weeks	12 weeks	24 weeks
Group A	0 ± 0	0 ± 0	0 ± 0	0 ± 0	0 ± 0
Group B	7.68 ± 2.59	9.57 ± 1.55	39.88 ± 5.65	48.30 ± 7.78	71.92 ± 6.39
Group C	3.15 ± 1.94^*^	4.07 ± 1.12^*^	4.88 ± 0.12^*^	7.37 ± 3.42^*^	34.66 ± 4.41^*^
Group D	3.36 ± 1.78^#^	5.70 ± 1.27^#^	26.56 ± 5.21^#□^	42.88 ± 8.59^□^	67.81 ± 3.57^□^
*F*	8.661	27.324	93.778	60.871	30.145
*P* value	< 0.05	< 0.05	< 0.05	< 0.05	< 0.05

Data are expressed as mean ± standard deviation. Group A had no difference in each time point and was not included in the statistical analysis. *P value < 0.05, group C compared with group B. ^#^*P* value < 0.05, group D compared with group B. □*P* value < 0.05, group C compared with group D

## Discussion

Among ceramic materials, TCP and HAP are two important components. HA is not easy to degrade but plays an important role in material stability and compressive capacity [19]. TCP reflects the strength and weakness of material biodegradation [20]. The ideal HA/TCP hybrid material can ensure that the material is firm and not deformed during the repair process, provide climbing effect for cell growth, and also take into account the growth of new tissue, without interference to the repair process, and ultimately not cause delayed healing or malformed healing. Lin et al. successfully reduced the high content of HA in the bovine bone by two calcinations and the addition of pyrophosphate and increased the TCP content [5]. On this basis, Xiu et al. applied it to pig bones and successfully prepared BCBB containing mainly TCP and HAP and confirmed its good cell compatibility [15]. Calcium sulfate (CS) is widely used as a bone graft binder and expander, which exhibits weak acidity after dissolution [21]. The weakly acidic environment is not conducive to cell growth, and this property is changed by physical and chemical treatment or addition of ions and functional groups, which increases the difficulty of operation and increases the production cost. BCBB solves this problem. In the body, BCBB materials can release a large amount of Ca+, P plasma, and related groups when they degrade, forming a slightly alkaline environment around the material, which can neutralize the slightly acidic environment formed by CS degradation [22].

In the present study, from the perspective of the repair process in animal experiments, the existence of SDF-1 accelerates the whole process of bone defect repair, achieving the goal of earlier repair and faster recovery, and the final repair effect is equivalent to the “gold standard” autologous bone no matter in X-ray performance, X-ray score, or new bone mass analysis, and satisfactory results were obtained. SDF-1 increases the local blood supply by promoting angiogenesis in the graft material [23], providing channels and nutrients for seed cell homing and proliferation, and promoting osteogenic differentiation by inducing MSCs to upregulate the expression of BMP-related proteins. For the osteoblasts that have been differentiated, it can enhance the osteogenesis by upregulating the expression of factors such as alkaline phosphatase and osteocalcin in the cells [24], while strengthening the BCBB-limited bone conduction ability at the same time, with good ability of bone induction. According to the results of tissue section in group D, in addition to the appearance of endochondral ossification, there was also an endochondral ossification trend at the graft material-tissue interface, which effectively proved that SDF-1 enhanced the osteogenesis effect of osteoblasts attached to the material surface. In addition, CS promotes chemotaxis of osteoprogenitor cells and promotes adhesion to promote final osteogenesis [25], and alkaline environment contributes to a cell differentiation [26], which provides an effective support for the enhancement of osteogenesis by SDF-1/BCBB scaffold, indicating that BCBB carrying SDF-1 has good bone repair ability.

MSCs as the basic cellular unit of embryologic bone formation are required for bone generation, bone repair, and remodeling [27]. MSC implantation has been widely applied for bone regeneration, bone repair, and metabolic bone disease in the clinic [28, 29]. SDF-1 is an important chemokine for recruitment and entrapment of MSCs [30]. In the present study, SDF-1 promoted the large bone repairment after BCBB scaffold implantation in rabbit models. Our findings may provide the basis of the application of SDF-1 in bone repairment, which could save the time for purification and culture-expandation of human MSCs.

Although a significant effect of SDF-1 on bone repairment after the bone graft has been found, there are some limitations in the present study. Firstly, the bone area was observed to evaluate the number of homing MSCs indirectly. The quantitative analysis of homing MSCs was not performed. Secondly, the effect of SDF1 on MSCs migration in vitro and homing in vivo with the application of SDF1 sustained-release system was not investigated. The related experiments are warranted in the near future.

## Conclusions

In conclusion, our study found that BCBB scaffold had a certain bone conduction capacity, and the BCBB scaffold carrying SDF-1 had improved bone conduction ability and possessed bone induction ability. In the case of carrying SDF-1, it can be used to repair large bone defects in a shorter time than simply using BCBB, and the ultimate repair effect of large bone defects is equivalent to that of autologous bone. However, there are still many problems to be studied and solved before clinical application. It is believed that this new bone tissue engineering scaffold can provide promising candidate treatment of bone injury with SDF-1 in the clinic.

## Data Availability

All data generated or analyzed during this study are included in this published article.

## Abbreviations

BCBB: Biphasic ceramic-like biologic bone
H&E: Hematoxylin-eosin
HAP: Hydroxyapatite
MSCs: Marrow mesenchymal stem cells
SDF-1: Stromal cell-derived factor-1
TCP: Tricalcium phosphate
